# Draft Genome Sequence of *Colletotrichum acutatum Sensu Lato* (*Colletotrichum fioriniae*)

**DOI:** 10.1128/genomeA.00112-14

**Published:** 2014-04-10

**Authors:** Riccardo Baroncelli, Surapareddy Sreenivasaprasad, Serenella A. Sukno, Michael R. Thon, Eric Holub

**Affiliations:** a University of Warwick, School of Life Sciences, Warwick Crop Centre, Wellesbourne, United Kingdom; b Centro Hispano-Luso de Investigaciones Agrarias (CIALE), Departamento de Microbiología y Genética, Universidad de Salamanca, Salamanca, Spain

## Abstract

In addition to its economic impact, *Colletotrichum acutatum sensu lato* is an interesting model for molecular investigations due to the diversity of host-determined specialization and reproductive lifestyles within the species complex. The pathogen *Colletotrichum fioriniae* forms part of this species complex and causes anthracnose in a wide range of crops and wild plants worldwide. Some members of this species have also been reported to be entomopathogenic. Here, we report the draft genome sequence of a heterothallic reference isolate of *C. fioriniae* (strain PJ7). This sequence provides a range of new resources that serve as a useful platform for further research in the field.

## GENOME ANNOUNCEMENT

Many species belonging to the genus *Colletotrichum* are causal agents of plant diseases, generally referred as anthracnose, in a wide range of hosts worldwide. Virtually every crop grown in the world is susceptible to one or more species of *Colletotrichum* (1). Many *Colletotrichum* species are characterized by a distinctive hemibiotrophic lifestyle. Members of the *Colletotrichum acutatum* species complex have a wide host range in both domesticated and wild plant species, and their capability to infect insects has also been described (2). Pathogenicity assays have shown that most isolates of the complex are not host specific (3–5). *C. fioriniae* (teleomorph: *Glomerella fioriniae*) strain PJ7 was isolated by Peter R. Johnston from infected strawberry (*Fragaria* x *ananassa*) fruit in the Auckland area, New Zealand, in 1988 (6, 7). The strain has been used as a reference strain for phylogenetic analyses of the *C. acutatum* species complex and for mating tests and pathogenicity assays (8, 9). The heterothallic mating capability of this strain has been demonstrated in laboratory experiments (8).

The genome sequence of *C. fioriniae* (*G. fioriniae*) strain PJ7 was obtained using Illumina mate-paired sequencing technology. Mate-paired reads of 50 bp and 70 bp (2.44 Gbp; average coverage, 49.7×) were assembled using Velvet (10). The contigs corresponding to the mitochondrial genome (mtDNA) and the rRNA-coding gene cluster were identified by BLASTn searches using Geneious R6. The mitochondrial genome was assembled into one scaffold using Geneious R6, with a total length of 29.868 Mbp and a G+C content of 30.10%. The mitochondrial DNA was inspected by tBLASTn searches to identify known conserved coding genes using *Colletotrichumgraminicola* mtDNA orthologs as the query sequences, resulting in the identification of 16 protein-coding genes and 29 tRNA-coding genes.

The draft nuclear genome of *C. fioriniae* consists of 1,108 sequence scaffolds with a total length 49.01 Mbp (*N*_50_, 137,254; *N*_90_, 38,253), 52.50% G+C content, and a maximum scaffold size of 596,408 bp. The completeness of the assembly was assessed using CEGMA version 2.4 (11), which estimated the genome sequence to be 98.39% complete. The nuclear genome was annotated using the MAKER pipeline (12), and tRNAscan was used to predict tRNAs (13). Overall, 13,759 protein-coding gene models and 317 tRNA-coding gene models were predicted in the nuclear genome. Of the protein-coding gene models, 11,039 (80.2%) are supported by protein and/or mRNA sequence evidence.

Analysis with WoLF PSORT (14) revealed that 2,203 predicted proteins (16.01% of the proteome) are secreted. Among those, 90 (4.09% of the secretome and 0.65% of the proteome) do not have any sequence similarity to proteins in public databases, based on BLAST searches. Such characteristics are typical of fungal effectors, which are proteins that have important roles in disabling the host defense system (15).

In this study, we generated the draft genome sequence from a member of the *C. acutatum* species complex. A number of distinct genetic groups within *C. acutatum sensu lato* were previously described (16), leading to recent disaggregation of the complex into 31 species (7). The sequence represents a new resource that will be useful for further research into the biology, ecology, and evolution of these key pathogens.

### Nucleotide sequence accession numbers.

This whole-genome shotgun project has been deposited in GenBank under the accession no. JARH00000000 (BioProject PRJNA233987). The version described in this paper is JARH00000000.1.
